# Almond Hull Extract Valorization: From Waste to Food Recovery to Counteract *Staphylococcus aureus* and *Escherichia coli* in Formation and Mature Biofilm

**DOI:** 10.3390/foods13233834

**Published:** 2024-11-28

**Authors:** Sara D’Arcangelo, Debora Santonocito, Luciano Messina, Valentina Greco, Alessandro Giuffrida, Carmelo Puglia, Mara Di Giulio, Rosanna Inturri, Susanna Vaccaro

**Affiliations:** 1 Department of Pharmacy, University “G. d’Annunzio” Chieti-Pescara, 66100 Chieti, Italy; sara.darcangelo@unich.it (S.D.); m.digiulio@unich.it (M.D.G.); 2 Department of Drug Sciences and Health, University of Catania, 95125 Catania, Italy; debora.santonocito@unict.it (D.S.); capuglia@unict.it (C.P.); 3 Fidia Farmaceutici SpA, Local Noto Unit Contrada Pizzuta, 96017 Noto, Italy; lmessina@fidiapharma.it (L.M.); svaccaro@fidiapharma.it (S.V.); 4 Department of Chemical Sciences, University of Catania, 95125 Catania, Italy; valentina.greco@unict.it (V.G.); alessandro.giuffrida@unict.it (A.G.)

**Keywords:** almond hull, eco-sustainability, food waste, biofilm, *S. aureus*, *E. coli*, food by-products, green extraction

## Abstract

The increase in food waste accumulation needs innovative valorization strategies that not only reduce environmental impacts but also provide functional applications. This study investigates the potential of almond hulls, an abundant agricultural by-product, as a source of bioactive compounds. For the first time, almond hull extract (AHE), was evaluated in terms of anti-adhesive and anti-biofilm activity against *Staphylococcus aureus* ATCC 29213 and *Escherichia coli* ATCC 9637. The extract was obtained by an optimized eco-friendly green technique using ultrasound-assisted extraction (UAE), and it was characterized for its main compounds by high-performance liquid chromatography–mass spectrometry (HPLC-MS) and nuclear magnetic resonance (NMR) analysis. Antimicrobial activity was evaluated on planktonic cells by minimum inhibitory/bactericidal concentration (MIC/MBC) and by 3-(4,5-dimethylthiazol-2-yl)-2,5-diphenyl tetrazolium bromide (MTT) assays. Afterward, AHE activity was evaluated against the bacterial sessile phase, both against in-formation and mature biofilm. Finally, the toxicity of the extract was tested on normal human adult cells (HDFa) by an MTT test. The principal active compounds present in AHE belong to the polyphenol group, in particular, the phenolic acid (Hydroxycinnammic sub-class) and, more significantly, the flavonoid class. The results showed that the extract has a relevant antimicrobial activity against the planktonic cells of both tested strains. Moreover, it significantly inhibited bacterial adhesion and promoted biofilm removal, highlighting its potential as a sustainable antimicrobial agent. The MTT test on human fibroblasts showed that the extract is not toxic for normal human cells. This research highlights how food waste valorization could have a high potential in the antimicrobial field.

## 1. Introduction

The food industry and food consumption generate around 40–60% of total municipal solid waste worldwide [1,2], with approximately 5 × 10^9^ tons from agro-forest and 5 × 10^8^ tons from food processing per year [3].

Conversely, by-products from the food-processing industry, including fruit pulp, peel, pomace, and seeds, as well as leaves, branches, flowers, and bark resulting from agro-industrial pruning operations, represent a significant source of bioactive compounds of considerable value, including proteins, lipids, and carbohydrates. These compounds have a great interest due to their potential applications in the biological and medical fields. Among these, polyphenols—a distinctive class of natural compounds—are already well-known for their antioxidant activity, as exemplified by their well-documented antioxidant properties [3,4]. For this reason, the food and agro-industries are investing in food waste management and valorization [4,5]. On the other hand, given the spread of antimicrobial resistance, research is also focusing on natural products, such as almond hulls, which may provide an alternative to antibiotics [6].

Among foods that produce waste and have recognized nutritional and health benefits, tree nuts are the most consumed worldwide. The tree nut family is the most abundant and consists of Brazil nuts, cashews, hazelnuts, macadamia nuts, pecans, pine nuts (technically a seed), almonds, pistachios, and walnuts, which are among the most consumed [7].

Consumption varies both between and within regions, with more than 40 regions using millions of hectares of land to grow and process tree nuts, with Europe in fifth place [8]. In Italy, almond cultivation is concentrated in the southeastern part of Sicily (Avola, Syracuse), where the production of the Pizzuta cultivar prevails [9]. The fruit is composed of a hull (the skin), a shell (the stone), and a seed (the kernel). After the dehulling process, almond seeds are consumed as dried fruit or as an ingredient in food products, thus generating a significant quantity of by-products represented by hulls and shells [10]. The shell biomass of almonds (*Prunus dulcis* Mill.) accounts for 35–62% of the total fresh weight of the fruit [11].

The almond husk, which constitutes the outer fibrous shell of the almond fruit, is characterized by a high concentration of bioactive compounds, including polyphenols belonging to the class of phenic acids (e.g., protocateic acid, p-coumaric acid, caffeic acid, and chlorogenic acid) and various compounds belonging to the different flavonoid subclasses, both in aglycone and glycosylated forms (e.g., catechin, epicatechin, quercetin, delphinidin, and cyanidin) [12]. These compounds provide antioxidant, anti-inflammatory, anticancer, and antimicrobial properties [12,13]. In particular, almond hull extract can inhibit the growth of strains belonging to the species *Staphylococcus aureus*, *Escherichia coli,* and *Pseudomonas aeruginosa* when they are in the planktonic phase [9]. However, there is a lack of studies regarding the ability to prevent or inhibit biofilm formation or facilitate removal.

The microbial world has developed extensive metabolic and protective mechanisms in response to external “selection pressure”, including pressure from antibiotics [14]. Different species, such as *E. coli*, *Klebsiella pneumoniae*, *S. aureus*, *Pseudomonas aeruginosa*, *Acinetobacter* spp., and *Enterococcus faecium*, are under surveillance by the European Centre for Disease Prevention and Control (ECDC) and represent a global concern due to the multidrug-resistant (MDR) phenotypes of certain strains [15,16]. In this context, the biofilm represents a threat that gives the producing strains a high degree of resistance to antimicrobial agents. Indeed, sessile bacterial cells release antigens and stimulate the production of antibodies that are no longer effective in killing the bacteria in biofilms. Biofilms can develop on both abiotic and biotic surfaces in a dynamic, stepwise process involving adhesion, growth, motility, and extracellular polysaccharide production [17,18].

A possible solution to counteract microbial biofilm, overcoming the problem of antibiotic resistance, is by using natural compounds [19].

Alkaloids, flavonoids, phenols, glycosides, steroids, saponins, and terpenoids are the most active secondary metabolites of plants [20]. In particular, the combination of phenolic compounds, such as catechin, protocatechuic acid, and vanillic acid, has been reported to have an inhibitory and killing effect against uropathogenic *E. coli* (UPEC), including biofilm-forming strains [21], and display antibacterial and antibiofilm effect also against staphylococcal strains [22].

Therefore, an optimized and eco-friendly method was used based on ultrasound-assisted extraction (UAE) to promote a suitable valorization of this by-product [23].

UAE uses sound waves to break down plant cell membranes, enhancing solvent penetration and bioactive compound release, showing many advantages when compared with conventional methods. The use of green solvents (water and ethanol) coupled with a short processing time results in high recovery yields [24,25,26].

Furthermore, short extraction times and low temperatures preserve the plant matrices from any degradation processes, such as peroxidation, oxidation, and hydrolysis, ensuring the high quality of the end products [27].

The use of a selected solvent mixture promotes the extraction of phenolic compounds such as protocateichuic acid, p-cumaric acid, caffeic acid, and chlorogenic acid [28]. The use of UAE ensured that the bioactive components remained stable and unaltered, preserving their integrity, which is crucial for an accurate evaluation of the antibacterial activity of the extracted compounds.

In this scenario, the aim of this study is to evaluate, for the first time, the ability of AHE to prevent biofilm formation and promote biofilm removal, providing a novel and sustainable approach to managing biofilm-related infections and fighting antimicrobial resistance. The study highlights the innovative use of almond by-products obtained from food waste and extracted through eco-friendly green methodology.

## 2. Materials and Methods

### 2.1. Almond Hull Extraction and Characterisation

#### 2.1.1. Chemicals

Ethanol (CAS 64-17-5, >99.8%), acetonitrile (CAS 75-05-8, ≥99.9%, gradient grade, suitable for HPLC), formic acid (CAS 64-18-6, MS grade), water (MS grade), and methanol-d4 (99.80 atom %D, CAS No. 811-98-3) were purchased from Sigma-Aldrich (Steinheim, Baden-Württemberg, Germany).

#### 2.1.2. Extraction

Almond fruits of *Prunus dulcis* Mill. (Pizzuta cultivar) were harvested in August–September in the eastern part of Sicily (Avola, SR, Italy). The hulls were separated manually and dried at 45 °C for 48 h in a drying oven (UM 400, Memmert, Büchenbach, Germany); weight loss was measured every 24 h. They were then crushed in a mechanical blender for 30 s. The obtained hull powder underwent an extraction process with an ethanol–water solution (80:20, *v*/*v*) in a 1:40 powder–solvent solution ratio (g/mL) in an ultrasonic device (UP 400 S, Dr. Hielscher GmbH, Stuttgart, Germany) for 22.5 min, maintaining the temperature (approx. 50 °C) in an ice bath. The extract was stirred for 3 h at room temperature and decanted overnight. After decantation, the precipitate was then further extracted with the same solvent mixture for 6 h under stirring and decanted again. The procedure parameters were used, as they represent an optimized methodology for the extraction of the total phenolic compounds from the almond hull [28,29,30]. Both supernatants were filtered through a paper filter and dried under a vacuum [27]. The extract was concentrated and dried using a rotavapor (Buchi R-300, Buchi, Milan, Italy) and stored at −20 °C until use.

#### 2.1.3. NMR Analysis

The crude reaction extract was analyzed using nuclear magnetic resonance (NMR). The dried crude extract was dissolved in 600 μL of deuterated methanol (MeOD) and then filtered through a nylon syringe filter with a 13 mm diameter and a 0.45 μm pore size. The prepared sample was placed in a 5 mm NMR tube and thoroughly mixed to ensure a uniform solution.

The ^1^H-NMR spectra were recorded on a Varian Unity Inova 500 MHz spectrometer. The experiments were performed in MeOD at 25 °C, and the chemical shifts are reported as δ (ppm) referenced to the resonance of the trimethyl silane (TMS) signal. The following parameters were applied: 16 scans, a 90° pulse (5 μs), and a relaxation delay (D1) of 2 s. The coupling constants (J) are given in Hz [31].

#### 2.1.4. HPLC-MS Analysis

All mass spectrometry (MS) experiments were performed using the LTQ XL mass spectrometer, equipped with an H-ESI II source, (ThermoFisher, San Jose, CA, USA) in full scan mode from *m*/*z* 150–2000 range. All measurements were performed in positive mode with a spray voltage of 2–3 kV. The capillary temperature was set to 250 °C, the capillary voltage to 20 V and the tube lens to 120 V. Ion trap collision-induced dissociation (CID) measurements were performed with a precursor ion selection window of 1–2 *m*/*z* with an activation time of 30 ms and helium as the collision gas. External calibration was performed using the Pierce LTQ ESI Positive Ion Calibration Solution. Data processing was performed using the FreeStyle Software ver. 1.6 SP1 (Thermo Fisher Scientific). The chromatographic analyses were performed according to previously established methods with minor modifications [32]. Briefly, the analysis was performed using a Thermo Scientific Vanquish High Performing Liquid Chromatography (HPLC) System (Thermo Scientific, Bremen, Germany) equipped with a Phenomenex (Torrance, CA, USA) Kinetex C-18 column (100 mm × 2.10 mm, 2.6 μm). The employed mobile phases were water–formic acid (A, 99.9:0.1, *v*/*v*) and acetonitrile–formic acid (B, 99.9:0.1, *v*/*v*). The gradient conditions used for LC separation were the following: 0 min 10% B and 60 min 100% B (held for 10 min). The flow rate was 0.25 mL/min. The injection volume was 5 μL, and the column was thermostated at 40 °C.

### 2.2. Antibacterial Activity of AHE

#### 2.2.1. Minimum Inhibitory Concentration and Minimum Bactericidal Concentration

The antimicrobial activity of AHE was evaluated using the broth microdilution method to determine the minimum inhibitory concentrations (MIC) and the minimum bactericidal concentration (MBC), in accordance with the Clinical & Laboratory Standards Institute (CLSI) guidelines [33].

The reference strains *S. aureus* ATCC 29213, which is a weak β-lactamase producer, and *E. coli* ATCC 9637, both purchased from the American Type Culture Collection (ATCC), were used for the assays. The AHE was dispensed into 96-well plates (Nunc, Euro Clone SpA, Life Sciences-Division, Milan, Italy) and diluted (ranging from 0.2 mg/mL to 100 mg/mL) in Mueller–Hinton broth (MHB, Oxoid, Milan, Italy). The strains were recovered from −80 °C and plated on Mueller–Hinton Agar (MHA, Oxoid, Milan, Italy). The inocula were prepared by inoculating individual colonies that had been pre-cultured overnight in MHB, in aerobic conditions with shaking at 120 rpm at 37 °C using the same incubation conditions. A 1:10 culture refresh was then performed for 2 h in MHB. After the incubation period, the bacterial cultures were diluted to achieve the optical density of 0.125 at 600 nm, which corresponds approximately to 5 × 10^7^ CFU/mL. Thus, the standardized broth cultures were diluted 1:100 in MHB to obtain a final concentration of 5 × 10^5^ CFU/mL. Into each well were added 100 µL of each broth culture, and the plates were incubated at 37 °C for 24 h.

As a positive control, strains were added to the extract-free medium to compare the bacterial growth levels. As a negative control, AHE diluted at tested concentrations was added to strain-free culture media.

The MBC values were determined by spreading 10 µL of the bacterial suspensions from the MIC value towards the highest concentrations of the substance on MHA plates.

#### 2.2.2. Effect on Bacterial Growth Curves

The effect of AHE on the growth curves of *S. aureus* ATCC 29213 and *E. coli* ATCC 9637 was also investigated by the microdilution method using 96-well plates. The plates were prepared following the same procedure as for the MIC determination, maintaining the same AHE and bacterial concentrations. Incubation was carried out in standardized conditions using the ELx808 Incubating Absorbance Plate Reader (Lonza, Basel, Switzerland). The optical density (OD) was read at 630 nm after regular shaking at a frequency of 30 min. The bacterial growth curves were obtained by plotting the mean OD_630_ values versus the time using Gen5 software (v.3.14 Agilent BioTek, Santa Clara, CA, USA).

After 6 h, the percentage of inhibition was calculated using the following formula:

(*CTR* − *treated sample*)/*CTR* × 100% (1)

where “CTR” is the positive control, while “treated sample” represents the bacteria treated with AHE at different concentrations.

#### 2.2.3. Effect on Bacterial Biofilm

**Figure 1:**
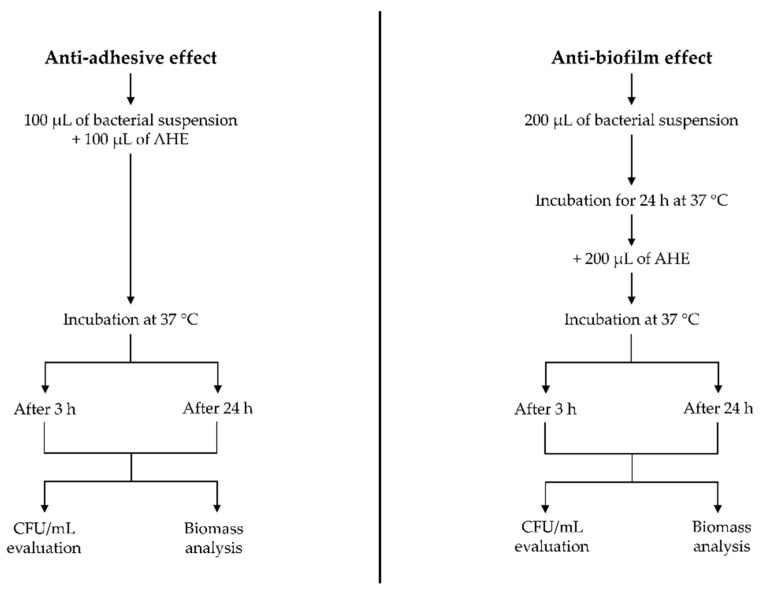
Test scheme of anti-adhesive and anti-biofilm evaluation against in-formation and mature biofilm, respectively. Tests were performed on both *S. aureus* ATCC 29213 and *E. coli* ATCC 9637.

The ability of AHE to affect the adhesion and to remove the mature mono-species biofilm was tested at different concentrations and times (3 h and 24 h) towards *S. aureus* ATCC 29213 and *E. coli* ATCC 9637, both biofilm-producer strains, as shown in Figure 1 [34,35]. The assays were carried out with 96-well flat-bottomed microtiter polystyrene plates, which represent an abiotic surface.

For the inoculation, the bacterial suspensions were prepared following the same procedure described for the MIC determination by using Tryptic Soy Broth (TSB, Oxoid, Milan, Italy) instead of MHB. After the refresh, the bacterial suspensions were diluted in TSB to achieve an optical density of 0.250 at 600 nm, which corresponds to 1.0 McFarland.

The ability to affect bacterial adhesion was evaluated using 3 different sub-MIC concentrations of AHE (1/2 MIC, 1/4 MIC, and 1/8 MIC) diluted in TSB [36].

On the other hand, the anti-biofilm activity of AHE against 24 h mature biofilm was analyzed using 2-fold, 4-fold, and 8-fold AHE MIC concentrations [37].

##### Anti-Adhesive Effect

For the anti-adhesive assay, 100 µL of AHE were added to each well and inoculated with 100 µL of the bacterial suspension. The plates were incubated at 37 °C in aerobic conditions for 3 h and 24 h. After the incubation time, each well was rinsed with sterile water, and the adherent cells were scraped off, resuspended in TSB, and plated on Tryptic Soy Agar (TSA, Oxoid, Milan, Italy) to determine the bacterial viable load through CFU/mL evaluation.

##### Anti-Biofilm Effect

To evaluate the anti-biofilm activity of the AHE, 200 µL of bacterial suspensions were added to each well and incubated at 37 °C in aerobic conditions for 24 h. Then, the planktonic bacteria were gently removed, and 200 µL of the AHE were added to each well and incubated for 3 and 24 h at 37 °C in aerobic conditions. The supernatant was removed, and each well was gently washed with sterile water. Then, they were scraped and resuspended in TSB and spread on TSA to determine the bacterial viable load through CFU/mL evaluation.

##### Biofilm Biomass Evaluation

Bacterial growth was evaluated also in terms of biomass quantification, as analyzed by spectrophotometer ELx808. Both for the anti-adhesive and the anti-biofilm assay, 3 wells for each treatment condition were only washed and used for biomass spectrophotometric evaluation using the following procedure. Briefly, 200 µL of 0.1% Crystal violet stain (Sigma Aldrich S.R.L, Milan, Italy) were added to each well after it was washed and dried at 25 °C. After 1 min, the stain solution was removed, and the wells were washed of the stain excess by gently adding 200 µL of sterile water. Then, the stained biomass within each well was eluted with 200 µL of ethanol, and the plate reading was performed at a wavelength of 570 nm.

#### 2.2.4. Metabolic Bacterial Activity

AHE’s capability to interfere with planktonic bacterial metabolic activity was evaluated through the 3-(4,5-dimethylthiazol-2-yl)-2,5-diphenyl tetrazolium bromide (MTT) assay [38]. Bacterial strains were grown in MHB and incubated at 37 °C for 24 h.

Overnight bacterial suspensions, grown in MHB at 37 °C in aerobic conditions, were standardized to an optical density (OD_600_ nm) of 0.18 after a 1:50 dilution was prepared as a working broth culture.

Bacterial suspensions were treated using on-scale MIC concentrations (1/8, 1/4, 1/2, MIC endpoint, and 2, 4, and 8-fold) of the AHE, ranging from 0.2 mg/mL to 12.5 mg/mL for *S. aureus* ATCC 29213 and from 1.6 mg/mL to 100 mg/mL for *E. coli* ATCC 9637. Non-treated bacterial samples were used as a positive control. The samples were incubated at 37 °C for 24 h. After incubation, bacterial suspensions were standardized to an optical density (OD_600_ nm) of 0.1. Then, after centrifugation for 60 s at 10,000 rpm, the supernatants were removed, and the pellets were resuspended in MHB. The optical density (OD_600_ nm) of each sample was read and recorded. Two hundred µL of each sample were transferred to a 1.5 mL empty tube, and 20 µL of MTT solution, previously equilibrated at 37 °C, were added, reaching a final concentration of 5 mg/mL. The tubes were incubated at 37 °C for 15 min. The supernatants were removed, and the pellets were resuspended in 2500 µL of Dimethylsulfoxide (DMSO, Sigma Aldrich S.R.L, Milan, Italy). The optical density was read at a wavelength of 550 nm (OD_550_ nm). The samples were analyzed in duplicate. The MTT assay was evaluated through the MTT reduction unit (MRU) using the following formula:

*MRU* = *OD_550_* × (2500 μL/220 μL) × (1000 μL*/*220 μL) × *DF*/*OD_600_* (2)

where OD_550_ is the absorbance read after the suspension of the MTT-treated pellet in DMSO, DF is the dilution factor used to standardize the bacterial suspensions, and OD_600_ is the absorbance read after the dilution of the pellet in MHB.

### 2.3. In Vitro Viability Assay on Human Dermal Fibroblast

In vitro viability was evaluated by an MTT assay on adult human dermal fibroblast primary cells (HDFa) purchased from American Type Culture Collection (ATCC^®^ PCS-201-012, Manassas, VA, USA) after treatment with AHE.

The HDFa were grown in a fibroblast basal medium (ATCC^®^ PCS-201-030), added with fibroblast growth kit serum-free (ATCC^®^ PCS-201-040) supplemented with Phenol Red (ATCC^®^ PCS-999-001) and with a Penicillin–Streptomycin–Amphotericin B solution (ATCC^®^ PCS-999-002). The cells were incubated at 37 °C, 5% CO_2_ for 48 h (CO-150, pbi New Brunswick Scientific, Enfield, CT, USA), followed by harvesting with Trypsin-EDTA (ATCC^®^ PCS-999-003) and Trypsin Neutralizing Solution (ATCC^®^ PCS-999-004).

The fresh medium was added to the cells to reach a final concentration of approximately 1.0 × 10^4^ cells/well, and 50 µL of the cell suspension were added to each well of the flat-bottomed 96-well plate and incubated at 37 °C and 5% CO_2_ for 24 h until 80% confluence was reached.

The cells were then treated with 50 µL of AHE prepared at various concentrations (from 0.2 mg/mL to 100 mg/mL) in a 1.0% RPMI/DMSO (1:1) solution. The plates were incubated for 72 h in the conditions described above.

The positive control was represented by extract-free cell suspension, while AHE diluted at the tested concentrations was added to the cell-free culture media as a negative control.

Then, the MTT solution (5 mg/mL) was added to each well and incubated for 3 h with shaking at 500 rpm. The absorbance at 570 nm was measured after the addition of 100 µL DMSO and incubation for 30 min with shaking at 500 rpm [39].

### 2.4. Statistical Analysis

All of the results were obtained from at least three independent experiments performed in triplicate, and they are expressed as means ± standard deviation. Values of *p* ≤ 0.05 were considered statistically significant. Differences among groups were evaluated with an ANOVA test.

## 3. Results

### 3.1. Almond Hulls Extraction and Characterisation

#### 3.1.1. Extraction and Weight Reduction

The extraction was performed using an ethanol–water solution (80:20, *v*/*v*) and a 1:40 powder–solvent solution ratio (g/mL), at 50 °C, since it was more suitable for obtaining a high yield and preserving the bioactive compounds [28,29,30]. In order to maximize the extraction yield, the obtained extract was subjected to two steps of stirring at 3 and 6 h, respectively [27].

The percentage of weight loss of the almonds after the drying process was 6.6% after one day and reached a maximum value of 12.6% after 3 days. After 2 and 3 days, the weight losses amounted to 3.9% and 2.1%, respectively. The weight loss was monitored until day 5, and no remarkable differences were observed compared to day 3 (Table 1). Starting from 1000 g trays, a final weight of 874 g was reached.

#### 3.1.2. NMR Analyses

**Figure 2:**
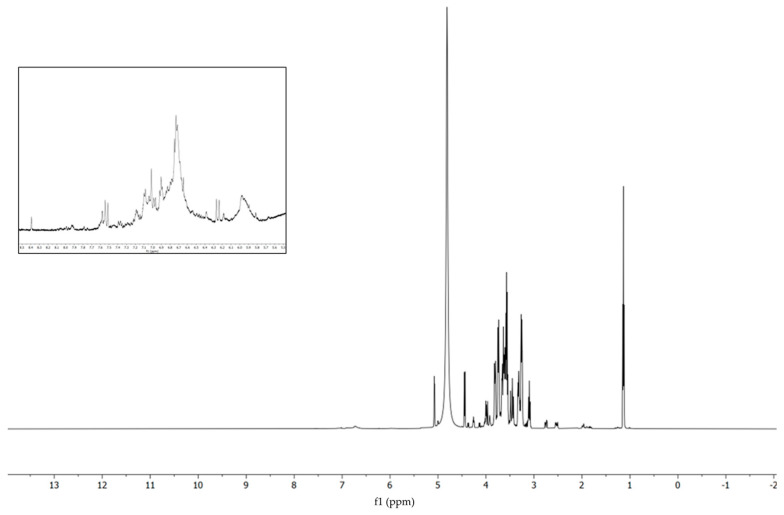
^1^H-NMR spectrum and magnification of aromatic field (insert).

The ^1^H-NMR spectrum (Figure 2) exhibits a characteristic profile, revealing multiple signals corresponding to carbohydrate moieties, in accordance with the extant literature on the structures of both simple and complex carbohydrates [40,41]. Notably, proton signals are observed at position 1 for the two anomeric forms of glucose (α: 5.07 ppm, β: 4.44 ppm), while the same type of signals corresponding to fructose and sucrose are found at 4.13 and 4.99 ppm, respectively. Between 3.0 and 4.5 ppm, signals attributable to the carbohydrate backbone are apparent, alongside methyl signals from methoxy groups in polyphenols. In the aliphatic region (0–2 ppm), the presence of only a few signals indicates a minimal lipid component extracted by this method. Moreover, the presence of chlorogenic acid is indicated by the distinctive signals, two ‘double doublets’, situated at 2.52 and 2.75 ppm, resulting from the diasterotopic protons at the sixth position of the aliphatic chlorogenic acid backbone and their correlation (as determined by the COSY spectrum Figure S1), with the signal at 4.25 ppm generated by the proton at the fifth position. Furthermore, the ^1^H-NMR spectrum displays (Figure 2, insert) signals associated with the aromatic rings of polyphenols (8-6 ppm). These signals exhibit relatively low intensity, which can be attributed to inefficient vibrational relaxation in an aqueous solvent, where the stacking interactions of aromatic rings are favored.

#### 3.1.3. HPLC-MS Analyses

Mass spectrometry scanning in negative electrospray ionization mode enabled the detection of all ions at *m*/*z* that have been previously reported in the literature [12,42,43,44]. This included the detection of phenolic acids (both benzoic and hydroxycinnamic class), such as protocateichuic acid, p-cumaric acid, caffeic acid, and chlorogenic acid. Furthermore, the principal aglycones of the flavonoid class, including catechin, epicatechin, and quercetin (Figure S2), were also identified.

**Figure 3:**
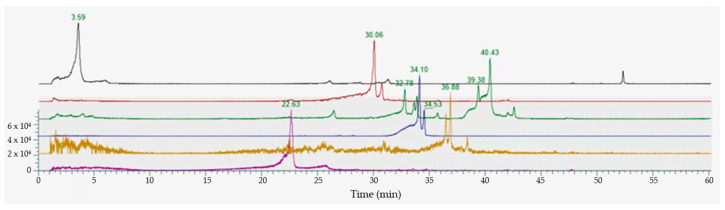
MRM profile of chlorogenic acid (black line); delphinidin-3-O-galactoside and delphinidin-3-O-glucoside (red line), cyanidin-3-O-glucoside (green line), petunidin-3-O-glucoside (blue line), peonidin-3-O-(p-coumaroyl-glucoside) (yellow line), peonidin-3-O-glucoside (purple line).

The analysis was performed in positive mode (Figure 3), which enabled the identification of glycated flavonoids, thereby confirming the data obtained by the NMR spectroscopy experiment concerning the abundant presence of the sugar component. The main compounds identified with their MRM transitions are provided below: chlorogenic acid (black line, *m*/*z* 355.3 and MS/MS: 192.7; 180.8); delphinidin-3-O-galacatoside (red line, *m*/*z*, 465.1 and MS/MS 303.1; 257.0; 229.2); delphinidin-3-O-glucoside (red line *m*/*z*, 465.1 and MS/MS 303.1; 257.0; 229.2); cyanidin-3-O-glucoside (green line, *m*/*z* 449.1 and MS/MS 287.1; 241.0); petunidin-3-O-glucoside (blue line, *m*/*z* 479.1 and MS/MS 317.1; 302.1; 274.1; 257.1); peonidin-3-O-(p-coumaroyl- glucoside) (yellow line, *m*/*z* 609.2 and MS/MS 301.1; 286.3; 258.2); and peonidin-3-O-glucoside (purple line, *m*/*z* 463.1 and MS/MS 301.1; 286.0; 258.2). The LC-MS/MS analysis revealed a characteristic elution pattern with chlorogenic acid eluting early (3.59 min) due to its high polarity, followed by anthocyanin glycosides in the 30–40 min range. Assuming similar ionization efficiency among anthocyanin glycosides, relative abundances indicated that delphinidin glycosides were the predominant anthocyanins (approximately 1.7-fold higher than other glycoside derivatives), while cyanidin, petunidin, and peonidin derivatives were present in similar proportions to each other. MS/MS fragmentation patterns showed characteristic neutral losses of 162 Da (corresponding to the dehydrated glycoside moiety, Doi:10.3390/ijms17050699) in all glycosylated compounds, particularly evident in the transitions of delphinidin–glycosides (*m*/*z* 465.1 → 303.1) and cyanidin–glucoside (*m*/*z* 449.1 → 287.1), providing complementary evidence to the NMR data for the glycosidic nature of these compounds.

### 3.2. Antibacterial Activity of AHE

#### 3.2.1. Minimum Inhibitory Concentration and Minimum Bactericidal Concentration

The results of the antibacterial activity of AHE against *S. aureus* ATCC 29213 and *E. coli* ATCC 9637 are shown in Table 2. The MIC concentration value was determined for both strains tested. The MIC for *S. aureus* ATCC 29213 was 7.8 times lower than that of *E. coli* ATCC 9637. Conversely, the MBC concentration value was also determined for *S. aureus* ATCC 29213 (3.2 mg/mL). However, for *E. coli* ATCC 9637, the MBC concentration value was higher than 100 mg/mL, which corresponds to the highest tested concentration.

#### 3.2.2. Effect on Bacterial Growth Curves

**Figure 4:**
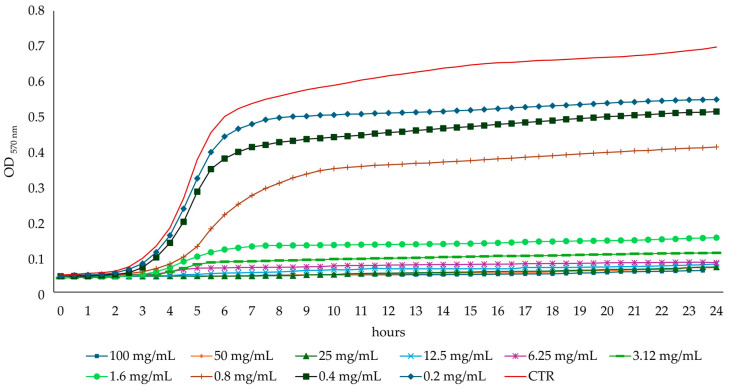
*S. aureus* ATCC 29213 growth curves in absence (CTR) or presence of different concentrations of AHE.

**Figure 5:**
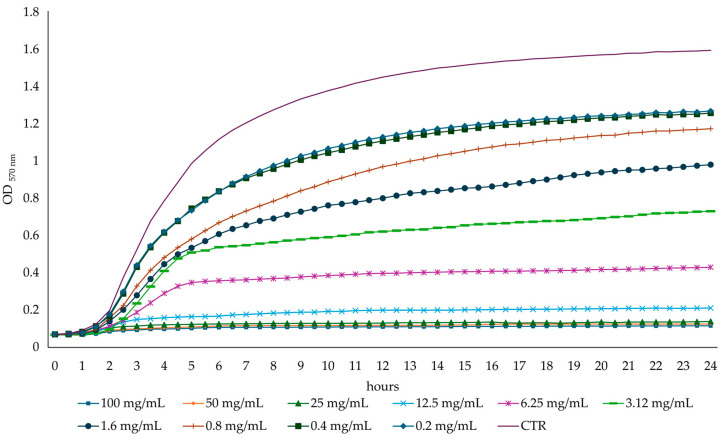
*E. coli* ATCC 9637 growth curves in absence (CTR) or presence of different concentrations of AHE.

The bacterial growth curves showed a dose-dependent effect of the AHE on bacterial growth and confirmed the previously determined MIC concentration values (Figure 4 and Figure 5). After 6 h of incubation, AHE at a concentration of 1.6 mg/mL inhibited the growth of *S. aureus* ATCC 29213 by 76% compared to the untreated control. Interestingly, the growth of *S. aureus* ATCC 29213 was also inhibited at sub-MIC concentrations (one-half, one-quarter, and one-eighth of the MIC value). The *S. aureus* ATCC 29213 growth was inhibited by 56%, 24%, and 11%, respectively, compared to the control.

After 6 h of incubation, the *E. coli* ATCC 9637 inhibition at the MIC value was about 85% compared to the untreated control, with a reduction of 68%, 52%, and 46% at one-half, one-quarter, and one-eighth of the MIC concentration value, respectively.

#### 3.2.3. Effect on Bacterial Biofilm

The effect of AHE on *S. aureus* ATCC 29213 and *E. coli* ATCC 9637 biofilm was investigated against both in-formation and mature biofilm to evaluate the anti-adhesive and anti-biofilm capabilities, respectively. Both experiments were performed at two different treatment times (3 h and 24 h).

##### Anti-Adhesive Effect

**Figure 6:**
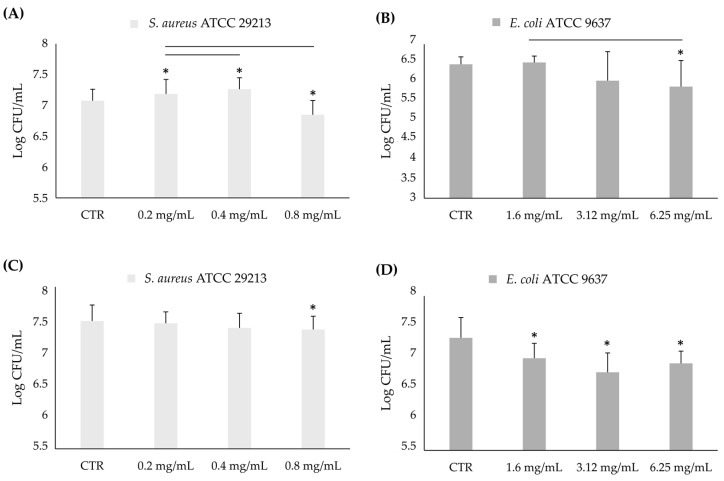
Log CFU/mL of *S. aureus* ATCC 29213 (**A**,**C**) and *E. coli* ATCC 9637 (**B**,**D**) in formation biofilms in presence of sub-MIC concentrations of AHE after 3 h (**A**,**B**) and 24 h (**C**,**D**). * Statistically significant with respect to the control. The lines indicate the intragroup significance.

Figure 6 shows the results of the anti-adhesive activity of AHE against the in-formation biofilm of *S. aureus* ATCC 29213 and *E. coli* ATCC 9637 after 3 h and 24 h.

A significant reduction in terms of CFU/mL with respect to the control was observed in the presence of one-half of the MIC concentration value for each strain (MIC *S. aureus* ATCC 29213 = 1.6 mg/mL; MIC *E. coli* ATCC 9637 = 12.5 mg/mL) after 3 h of incubation (Figure 6A,B). A decrease of 39% and 46% compared to the untreated control was observed for *S. aureus* ATCC 29213 and *E. coli* ATCC 9637 in terms of the viable percentage, corresponding to a reduction of 0.27 and 0.58 logarithms.

After 24 h (Figure 6C,D), *S. aureus* ATCC 29213 growth was influenced in the presence of 0.8 mg/mL of AHE, with a reduction of 27% of CFU/mL, corresponding to 0.13 logarithms. *Escherichia coli* ATCC 9637 growth was inhibited in a more pronounced manner at all of the tested concentrations, with significant reductions of 58%, 71%, and 67% CFU/mL with respect to untreated (0.33, 0.56, 0.41 logarithms, respectively) at one-eighth, one-fourth, and one-half of the MIC concentration value (corresponding to 1.6, 3.12, and 6.25 mg/mL).

##### Anti-Biofilm Effect

The anti-mature biofilm activity of the AHE was evaluated against *S. aureus* ATCC 29213 and *E. coli* ATCC 9637 after 3 h and 24 h of treatment with the natural extract.

**Figure 7:**
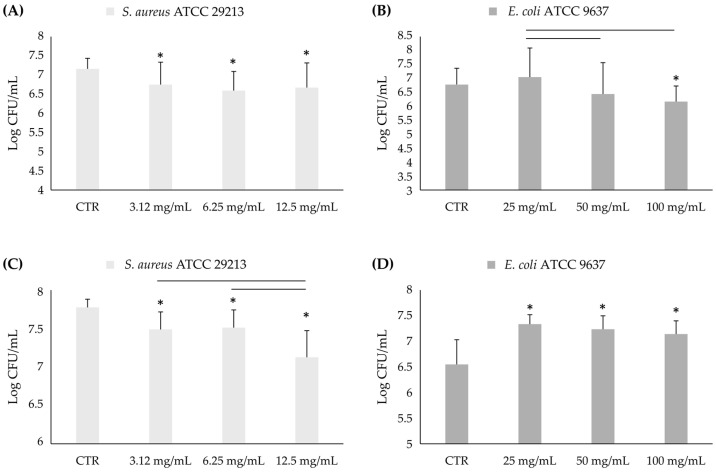
Log CFU/mL of *S. aureus* ATCC 29213 (**A**,**C**) and *E. coli* ATCC 9637 (**B**,**D**) mature biofilms in presence of over-MIC concentrations of AHE after 3 h and 24 h. * Statistically significant with respect to the control. The lines indicate the intragroup significance.

The AHE significantly reduces *S. aureus* ATCC 29213 mature biofilm in comparison to the untreated control at all tested concentrations and treatment times (3 h and 24 h). In detail, after 3 h, the reduction rates are 37%, 57%, and 39% (corresponding to 0.41, 0.57, and 0.49 logarithms reduction, respectively) at 2-, 4- and 8-fold MIC concentration values (Figure 7A).

After 24 h, the reduction rates are 44%, 40%, and 70% (corresponding to 0.29, 0.26, and 0.65 logarithms reduction, respectively) at 2-, 4- and 8-fold MIC concentration values (Figure 7C). AHE significantly decreased the *E. coli* ATCC 9637 biofilm only at the highest tested concentration (100 mg/mL, corresponding to 8-fold MIC concentration values) after 3 h (Figure 7B). Conversely, after 24 h, it showed an increase in the CFU/mL of *E. coli* ATCC 9637 at all concentrations (Figure 7D).

##### Biofilm Biomass Evaluation

**Figure 8:**
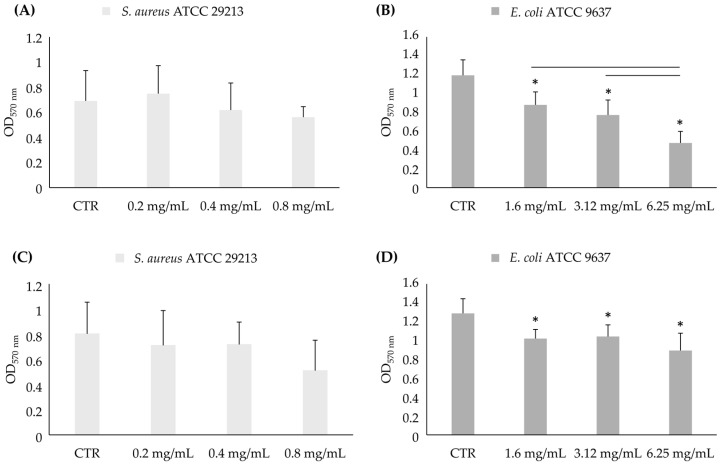
Biomass analysis of in-formation biofilm of *S. aureus* ATCC 29213 (**A**,**C**) and *E. coli* ATCC 9637 (**B**,**D**) in biofilms formation in presence of sub-MIC concentrations of AHE after 3 h and 24 h. * Statistically significant with respect to the control. The lines indicate the intragroup significance.

The results regarding the biofilm biomass against the in-formation biofilm (Figure 8) show a significant reduction with respect to the control only for *E. coli* ATCC 9637 at all concentrations, both after 3 and 24 h (Figure 8B,D). In particular, there was a decrease in biomass formation of 26%, 35%, and 60% after 3 h and 21%, 19%, and 31% after 24 h at one-eighth, one-fourth, and one-half of the MIC value, respectively.

**Figure 9:**
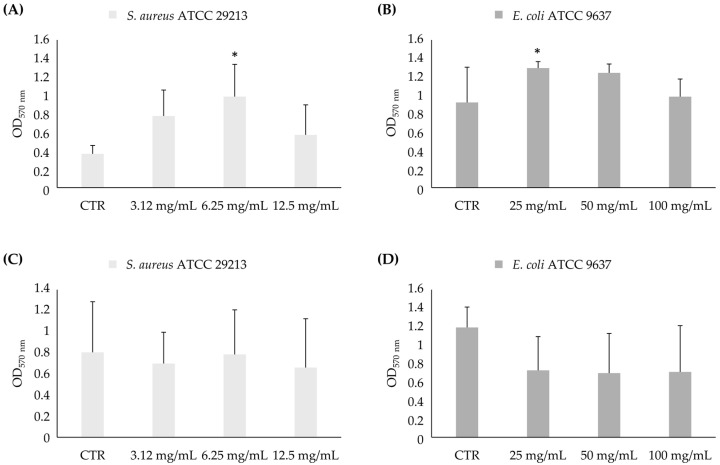
Biomass analysis of mature biofilm of *S. aureus* ATCC 29213 (**A**,**C**) and *E. coli* ATCC 9637 (**B**,**D**) in presence of over-MIC concentrations of AHE after 3 h and 24 h. * Statistically significant with respect to the control. The lines indicate the intragroup significance.

The biofilm biomass analysis of the mature biofilm showed a significant increase in the *S. aureus* ATCC 29213 mature biofilm at 6.25 mg/mL (4-fold MIC) and 25 mg/mL (2-fold MIC) for *E. coli* ATCC 9637 after 3 h (Figure 9).

#### 3.2.4. Metabolic Bacterial Activity

**Figure 10:**
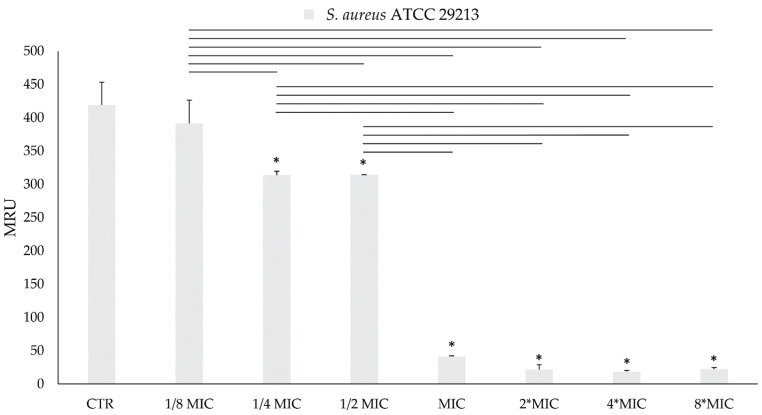
MTT (3-(4,5-dimethylthiazol-2-yl)-2,5-diphenyl-2H-tetrazolium bromide) reduction activity of planktonic cells of *S. aureus* ATCC 29213 after treatment with AHE at different concentrations. * Statistically significant with respect to the control. The lines indicate the intragroup significance.

**Figure 11:**
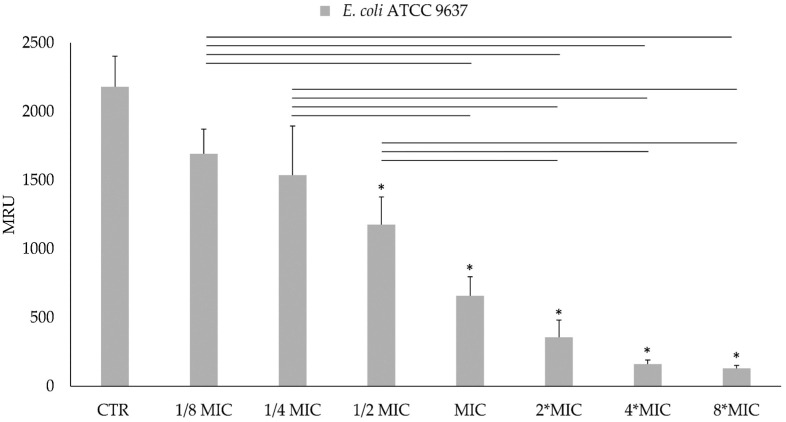
MTT (3-(4,5-dimethylthiazol-2-yl)-2,5-diphenyl-2H-tetrazolium bromide) reduction activity of planktonic cells of *E. coli* ATCC 9637 after treatment with AHE at different concentrations on * Statistically significant with respect to the control. The lines indicate the intragroup significance.

The MTT assay performed on bacterial planktonic cells shows a dose-dependent growth inhibition for both of the tested bacteria, as can be noticed in Figure 10 (*S. aureus* ATCC 29213 viability) and in Figure 11 (*E. coli* ATCC 9637 viability). In particular, for *S. aureus* ATCC 29213, the cell viability reduction at the MIC value (1.6 mg/mL) was more than 90%, while there was a reduction of 70% in *E. coli* ATCC 9637 viability at the MIC value (12.5 mg/mL). The highest reduction rate was registered at 8-fold MIC for both bacteria. In this condition, *S. aureus* growth decreased by 95%, while *E. coli* growth decreased by 94%. The results of the MTT reduction activity are expressed as MRU.

### 3.3. In Vitro Viability Assay on Human Dermal Fibroblast

**Figure 12:**
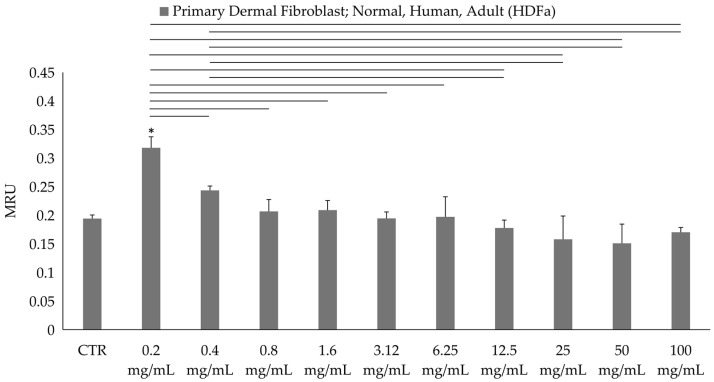
MTT (3-(4,5-dimethylthiazol-2-yl)-2,5-diphenyl-2H-tetrazolium bromide) results of AHE at different concentrations on primary dermal fibroblast; normal, human, adult (HDFa). * Statistically significant with respect to the control. The lines indicate the intragroup significance.

The viability of HDFa was measured through the evaluation of metabolic activity. The MTT assay showed no significant differences between the CTR (non-treated fibroblast) and the treated samples at all of the tested concentrations, except for the lowest concentration (0.2 mg/mL) in which the metabolic activity significantly increased (Figure 12).

## 4. Discussion

Food waste has become a critical global challenge, accounting for about one-third of all food produced annually. For this reason, innovative management strategies are requested in order to create opportunities of food recovery and valorization [3,45].

Natural extracts from food waste, such as pomegranate peel, citrus peel (tangerines and oranges), almond hull, fruit/vegetable leaves, and the shells of coconut and other fruits, are rich in polyphenols, antioxidants, vitamins, and other beneficial compounds.

Vegetable materials, which may include plant materials, algae, macroscopic fungi, or combinations thereof, are defined by the US Food and Drug Administration (FDA) as botanical drug products. These products may be available in various pharmaceutical forms, such as solutions, powders, tablets, capsules, and elixirs, and may be administered by topical application or injection.

The Centre for Drug Evaluation and Research (CDER) has published a Botanical Drug Development Guidance for Industry to take these characteristics into account and to facilitate the development of new therapies from botanical sources.

To date, four botanical products have met the Botanical Guidance’s definition of a botanical drug product. Sinecatechins, crofelemer, and birch triterpenes are examples of FDA approved botanical drugs. Anacaulase-bcdb is another plant-derived product that obtained the biologic licence. Moreover, some botanical drugs, including cascara, psyllium, and senna, are included in the review of over-the-counter (OTC) drugs.

These plant-derived food wastes are increasingly recognized for their antimicrobial properties, which can prevent bacterial growth acting both on the planktonic and sessile phases [46]. Rich in bioactive compounds, such as polyphenols and flavonoids, plant-derived food wastes can inhibit harmful pathogens, including those found on the skin. For example, in a recent study, pomegranate peel extract was shown to reduce the biofilm formation of *S. aureus* without disturbing the beneficial microbial community in mono-species biofilm [36].

This green approach not only reduces waste but also supports a circular economy, creating skin care products that benefit both consumers and the environment [47].

Almonds are one of the most consumed tree nuts worldwide and consist of four portions: the kernel, middle shell, outer green shell cover (almond hull), and a thin leathery layer (brown skin seed coat) [7]. The hull accounts for three-fourths of almond waste production [48]. Each year, for every unit of almond kernels produced, there are approximately 1.6 units of almond hulls generated, and for this reason, the hull represents the principal almond by-product [49]. Moreover, they are rich in bioactive compounds and are extensively studied for their antioxidant activity. However, effective extraction methods open the route to other applications such as antimicrobial, anti-inflammatory, antiviral, anti-carcinogenicity, and antiaging [50].

Almond hull extract was obtained by an ultrasound-assisted extraction (UAE), an eco-friendly technique capable of disrupting plant cell membranes through the use of sound waves. This allows for enhanced solvent penetration and improves the quality of the final products. The UAE method has been chosen for the extraction of bioactive compounds from almond hulls. This choice was dictated by the numerous advantages of this technique, such as high extraction yields and the quality of the final products, as this method is able to maximize the extraction of bioactive components, preserving their health-promoting activity. First, the almond hulls were pulverized in a mechanical blender for 30 s in order to obtain a granulometry suitable for extraction. Then, the extraction process was performed using UAE coupled with stirring according to our previous study [27], slightly modified. In particular, the process was carried out at 50 °C, in order to preserve the plant matrix, using an ethanol–water mixture (80:20 *v*/*v*). As reported in the literature [28], this solvent ratio has proven to be more suitable for the extraction of phenolic compounds from almond hulls.

Although several studies report the antioxidant activity of AHE, there are few studies regarding the antimicrobial potential, and there are no studies regarding the antibiofilm activity against microorganisms.

In this study, the HPLC analysis of AHE confirmed protocatechuic acid, acid p-coumaric, caffeic acid, chlorogenic acid, catechin, epicatechin, and quercetin as the main detected phytochemicals.

These bioactive compounds present in the almond hull were reported to exhibit antimicrobial potential [51,52]. The antibacterial mechanism of polyphenolic compounds is associated with their ability to form hydrogen bonding with cell membrane proteins, destroying electron transport chains and membranes [53]. On the other hand, chlorogenic acid increases the outer and plasma membrane’s permeability [54].

In particular, as reported in the literature, catechins exert antibacterial activity against *E. coli*, showing an MIC of 1–2 mg/mL and an MBC of 2–4 mg/mL. Moreover, they were able to downregulate (≥60%) *acrA*, the gene related to the expression of biofilm [55]. Protocatechuic acid is also a promising natural compound with antimicrobial activity against Gram-positive and Gram-negative bacteria [56,57,58], showing a synergistic effect with antibiotics [59].

As reported in recent works, AHE has the capability to inhibit the microbial growth of different bacteria, such as *S. aureus* and *E. coli* [28,60].

Similarly, the results of this study showed inhibitions of bacterial growth at 1.6 and 12.5 mg/mL for *S. aureus* and *E. coli,* respectively, confirming the antimicrobial properties of the almond hull.

The anti-adhesive activity of AHE against *S. aureus* and *E. coli* highlights the different impact of sub-MIC concentrations of the extract on biofilm formation over time. The significant reduction of adhered cells observed after 3 h at one-half MIC for both strains (0.8 mg/mL and 6.25 mg/mL for *S. aureus* ATCC 29213 and *E. coli* ATCC 9637, respectively) suggests that AHE effectively affects the early stage of biofilm development, particularly by preventing bacterial adhesion, a key step in biofilm formation. The moderate reduction in *S. aureus* biofilm formation compared to *E. coli* after 3 h may reflect differences in the biofilm initiation processes between Gram-positive and Gram-negative bacteria [61]. After 24 h, *S. aureus* biofilm formationwas significantly reduced at 1/2 MIC value, while *E. coli* showed a more noticeable reduction in biofilm formation at all of the tested concentrations. This suggests a more sustained anti-adhesive effect on Gram-negative bacteria, probably affecting the lipopolysaccharide (LPS), which is crucial for bacterial adhesion on surfaces during the initial stage of biofilm formation [62].

Regarding the anti-biofilm activity, the AHE showed a significant reduction for *S. aureus* against mature biofilm at all of the tested concentrations, both after 3 and 24 h. *E. coli* biofilm was reduced only at an 8-fold MIC concentration after 3 h. The observed anti-biofilm activity of AHE suggests that this natural extract may have a different effect on Gram-positive and Gram-negative biofilms, probably due to the different auto-inducer peptides produced by the quorum-sensing system [63].

Regarding the in-formation biofilm biomass, the significant reduction observed only for *E. coli* at all tested concentrations, both at 3 and 24 h, further underscores the AHE ability to interfere with biofilm matrix development in Gram-negative bacteria, suggesting that AHE impacts not only bacterial adhesion but also the production of extracellular polymeric substances (EPS).

The MTT metabolic assay against bacteria showed a dose-dependent reduction in planktonic cell viability for both *S. aureus* and *E. coli*, reaching over 94% growth reduction for both bacteria. This suggests that AHE has stronger antibacterial effects at higher concentrations, likely due to increased membrane disruption or metabolic interference.

The findings of the MTT metabolic assay using in vitro human cells indicate that the extract is generally non-cytotoxic to human dermal fibroblasts. However, at the lowest concentration (0.2 mg/mL), a significant increase in metabolic activity was observed. This suggests a potential stimulatory effect on cell metabolism at lower doses, possibly promoting cell growth or proliferation.

## 5. Conclusions

In conclusion, this study demonstrates for the first time the potential of almond hulls as a valuable, sustainable resource to fight biofilm-associated infections. The strength of this work is the use of a food waste that is largely produced in the food industry, as it represents an abundant agricultural residue. Moreover, by using this by-product, the use of a local product was valued. These findings support the possibility of further study of AHE in preclinical and clinical studiesfor topic application. By combining food waste valorization with an innovative antimicrobial strategy, AHE represents a step toward both improved public health outcomes and environmental sustainability.

## Figures and Tables

**Table 1:** Weight and reduction rate of almond hull after drying at 45 °C for 3 days. Reductions are expressed as percentage.

Days	Weight (g)	Reduction than “Day 0” (%)	Reduction than the Previous Day (%)
0	1000		
1	934	6.6	
2	895	10.5	3.9
3	874	12.6	2.1

**Table 2:** Minimum inhibitory concentration (MIC) and minimum bactericidal concentration (MBC) values of AHE against reference strains. Concentrations are expressed in mg/mL.

Strains	MIC	MBC
*S. aureus* ATCC 29213	1.6	3.2
*E. coli* ATCC 9637	12.5	>100

## Data Availability

The original contributions presented in the study are included in the article/Supplementary Materials; further inquiries can be directed to the corresponding author.

## Supplementary Materials

The following supporting information can be downloaded at https://www.mdpi.com/article/10.3390/foods13233834/s1: Figure S1: gCOSY spectrum of crude extract in MeOD; Figure S2: Chromatogram of crude extracted in ESI(-)MS mode.
