# Supplementary material for: Almond Hull Extract Valorization: From Waste to Food Recovery to Counteract Staphylococcus aureus and Escherichia coli in Formation and Mature Biofilm
Source: Foods. 2024 Nov 28;13(23):3834. doi: 10.3390/foods13233834 (PMC11640323; doi:10.3390/foods13233834)
Supplement: Supplementary file 1 [file foods-13-03834-s001.zip › foods-3292075-supplementary.pdf]

Almond Hull Extract Valorization: from Waste to Food Recovery to Counteract *Staphylococcus aureus* and *Escherichia coli* in Formation and Mature Biofilm

Index

Figure S1 ……………………………………………………………………………………..gCOSY of crude extract

Figure S2…………………………………………………………………………….HPLC-ESI(-)MS of crude extract

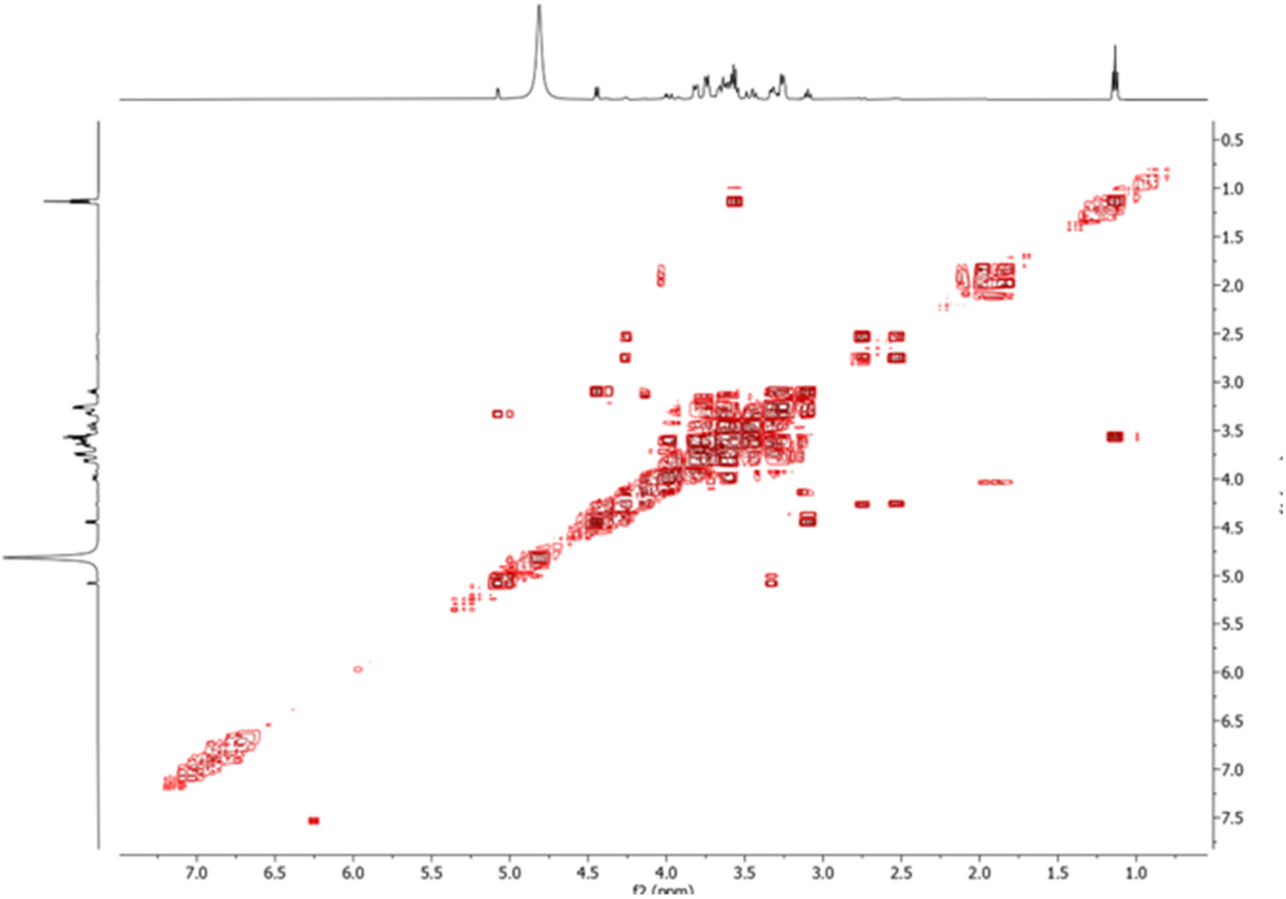

Figure S1. gCOSY spectrum of crude extract in MeOD.

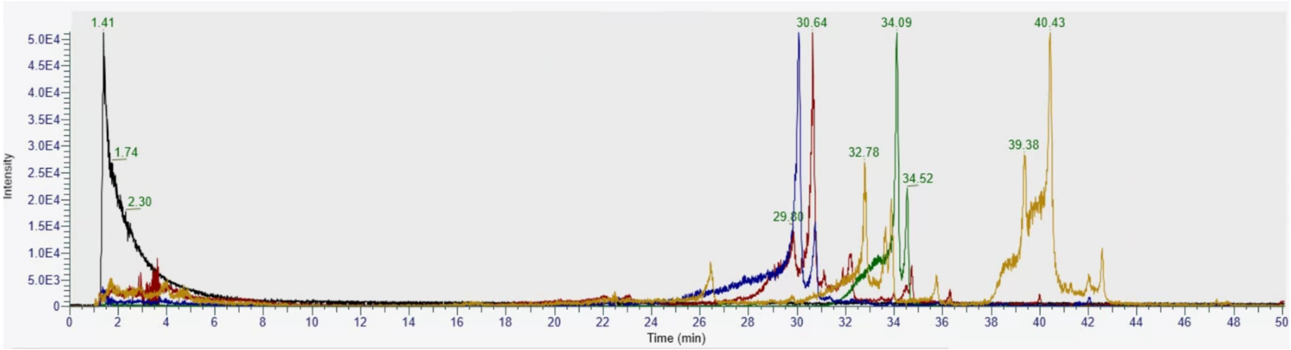

Figure S2. Chromatogram of crude extracted in ESI(-)MS mode.
